# Short Hypoxia Does not Affect Plasma Leptin in Healthy Men under Euglycemic Clamp Conditions

**DOI:** 10.1155/2009/270698

**Published:** 2009-06-01

**Authors:** Andre Schmoller, Michaela Voss, Hartmut Gehring, Sebastian Rudolf, Ulrich Schweiger, Bernd Schultes, Kerstin M. Oltmanns

**Affiliations:** ^1^Department of Psychiatry and Psychotherapy, University of Luebeck, D-23538 Luebeck, Germany; ^2^Department of Anaesthesiology, University of Luebeck, D-23538 Luebeck, Germany; ^3^Interdisciplinary Obesity Center, St. Gallen, CH-9400 Rorschach, Switzerland; ^4^Department of Neuroendocrinology, University of Luebeck, D-23538 Luebeck, Germany

## Abstract

Leptin is involved in the endocrine control of energy expenditure and body weight regulation. Previous studies emphasize a relationship between hypoxic states and leptin concentrations. The aim of this study was to investigate the effects of acute hypoxia on leptin concentrations in healthy subjects. We examined 14 healthy men. Hypoxic conditions were induced by decreasing oxygen saturation to 75% for 30 minutes. Plasma leptin concentrations were determined at baseline, after 3 hours of euglycemic clamping, during hypoxia, and repeatedly the following 2.5 hours thereafter. Our results show an increase of plasma leptin concentrations in the course of 6 hours of hyperinsulinemic-euglycemic clamping which may reflect diurnal rhythmicity. Notwithstanding, there was no difference between levels of leptin in the hypoxic and the normoxic condition (*P* = .2). Since we did not find any significant changes in leptin responses upon hypoxia, plasma leptin levels do not seem to be affected by short hypoxic episodes of moderate degree.

## 1. Introduction

Hypoxia is suggested to play an important role in the pathogenesis of endocrine and metabolic dysregulation in obesity and the metabolic syndrome [1]. We could previously demonstrate, based on an experimental human in vivo design established in our group, that 30 minutes of oxygen desaturation to 75% are sufficient to induce features of the metabolic syndrome such as glucose intolerance [2, 3] and respective changes in sympathetic activation [2, 3], resting energy expenditure [2, 3], and vascular endothelial growth factor (VEGF) levels [4], a hormone which has been assumed to play a role in the pathogenesis of the metabolic syndrome [5]. 

Another hormone centrally involved in body weight regulation and thereby the development of the metabolic syndrome is leptin [6]. Leptin is synthesized mainly, but not exclusively, by white adipose tissue sending a feedback signal to the brain about the amount of peripheral fat storages. The secretion of leptin underlies various influencing factors. Recent literature indicates that circulating leptin concentrations are possibly increased by hypoxic conditions in humans, suggesting a link to the obstructive sleep apnea syndrome (OSAS), a disease characterized by recurrent night apneic periods, which is strongly associated with obesity [7]. Indeed, as compared to healthy controls, OSAS patients show elevated leptin concentrations which return to normal after elimination of night apneas by continuous positive airway pressure (CPAP) therapy [8]. However, it remains unclear if high leptin levels in these patients are related to hypoxia, to hormonal interactions, or to the frequently increased body mass in OSAS. At high altitude (3000–4000 m), that is, under constant hypoxic conditions, increased [9, 10] as well as decreased leptin concentrations [11] have been reported in humans as compared to sea level. Potential confounders of leptin secretion in this setting are physical strain, nutritional differences, or physical stress upon ascension. Previous studies using cell cultures [12–14] and animal models have provided [15] a contradicting evidence for an impact of hypoxia on leptin concentrations. 

In order to clarify if hypoxia affects circulating leptin concentrations in humans, we experimentally induced a short hypoxic period as compared to normoxic condition in healthy normal weight participants according to our established study design which has been demonstrated to induce clinically relevant changes in energy and hormone metabolism [2, 3]. Oxygen saturation of 75%, as applied in our study, corresponds to a physiological state at approximately an altitude of 3500–4000 m. To control for common biases known to influence leptin metabolism, we performed our study at rest. Moreover, our group has previously shown that the glucose metabolism is a main determinant of leptin secretion [16, 17]. Each subject, therefore, underwent an euglycemic glucose clamp procedure to ensure stable circulating glucose and insulin concentrations throughout the experiments to control for these influencing factors on plasma leptin levels. Additionally, sympathetic activation as evaluated by circulating epinephrine and norepinephrine concentrations was monitored.

## 2. Methods and Procedures

### 2.1. Subjects

Experiments were performed including 14 healthy men aged 20–25 years. Exclusion criteria were overweight (body mass index (BMI) >25 kg/*m* ^2^), chronic or acute illness (particularly respiratory diseases), alcohol or drug abuse, smoking, competitive sports, exceptional physical or mental stress, and current medication of any kind. Each participant gave a written informed consent, and the study was approved by the local ethics committee.

### 2.2. Experimental Design

Subjects participated in a hypoxic and a normoxic session separated by an interval of at least 4 weeks. Hyperinsulinemic-euglycemic clamp procedures were performed during the entire experimental testing. A detailed clamping procedure has been described previously [2, 17]. Subjects were blinded for conditions. 

On the days of experimental testing, subjects reported to the medical research unit at 11:00 h after an overnight fast of at least 12 hours. After 3 hours of clamping, hypoxia was induced for 30 minutes by decreasing oxygen saturation to 75%. On the normoxic control condition, oxygen saturation was held at the physiological level of 98%. We assessed the course of leptin concentrations at baseline and repeatedly 3 hours after induction of hypoxia according to leptin's half life in normal weight men of about 3 hours by punctional determination [18]. During induction of hypoxia and also during the normoxic control period, participants breathed at normobaric conditions through a tightly fitting face mask connected to a Trajan 808 fresh gas supply (Draeger Medical Technology, Luebeck, Germany), using a valveless high-flow system between 14 and 17 l/min. Inspired oxygen fraction (*F*_*i*_ *O*_2_) was varied by adjusting oxygen and nitrogen. Oxygen saturation was continuously measured by pulse oxymeter and was decreased to 75% within approximately 5 minutes. After 30 minutes, oxygen saturation was quickly normalized. Catecholamine levels were monitored at baseline and every 7.5 minutes during hypoxia (versus control).

### 2.3. Assays

All blood samples were immediately centrifuged and the supernatants stored at −24°C until assay. Leptin concentrations were measured by ELISA (Diagnostic Systems Laboratories, Sinsheim, Germany; interassay coefficient of variation (CV) 4.4%, intra-assay CV 3.8%). Serum insulin was measured by radioimmunoassay (Pharmacia Insulin RIA 100, Pharmacia Diagnostics, Uppsala, Sweden; inter-assay coefficient of variation (CV) < 5.8%, intra-assay *CV* < 5.4%). Blood glucose concentrations were monitored at 5-minute intervals throughout the experiment (Glucose Analyser, Beckman Coulter, Inc. Munich, Germany). High pressure liquid chromatography was applied to measure plasma epinephrine (intra-assay *CV* < 2.9%; inter-assay *CV* < 4.2%) and norepinephrine levels (intra-assay *CV* < 2.6%; inter-assay *CV* < 3.9%, Chromosystems, Munich, Germany).

### 2.4. Statistical Analysis

Values are presented as mean values ±SEM. Analyses of variance (ANOVA) for repeated measurements, including the factors “session” (hypoxia versus normoxia) and “time” (time points of data collection) were implemented and corrected according to the Greenhouse-Geisser procedure. The interaction effect of these two factors was termed “session by time.” The area under the curve (AUC) was calculated from 6 hours leptin short-profiles to determine overall differences in leptin secretion between the two sessions. In addition, student's paired *t*-tests were performed to compare hormone concentrations at single time points. A *P*-value <.05 was considered significant.

## 3. Results

### 3.1. Oxygen Saturation, Serum Insulin, Plasma Glucose, and Catecholamines

**Figure 1:**
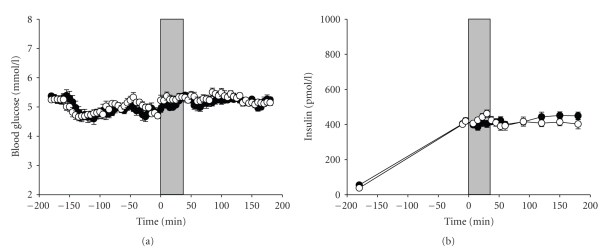
Mean (±SEM) blood glucose concentrations (a) and plasma insulin (b) in the hypoxic (white circles) and the control (black circles) condition. Gray area marks the time of hypoxic/normoxic intervention. The time course describes the period before and after the hypoxic intervention was initiated (Time point 0).

Upon induction of hypoxia, oxygen saturation decreased over a period of about 5 minutes and was held stable at a plateau of 74 ± 2%. Oxygen saturation (corresponding to 35–41 mmHg PaO2) during the corresponding period under the normoxic condition remained at a mean level of 98 ± 2%. Determination of plasma glucose concentrations and serum insulin confirmed that respective blood levels remained constant after having reached the equilibrium and did not differ between hypoxic and normoxic conditions throughout the study (Figure 1). Also, as already published [3], norepinephrine concentrations did not differ significantly between the two sessions (*P* = .3) whereas epinephrine levels were significantly higher under the hypoxic condition as compared with the control session (*P* < .01). The glucose infusion rates were significantly lower during the hypoxic as compared to the normoxic period [2].

### 3.2. Plasma Leptin Concentrations

**Figure 2:**
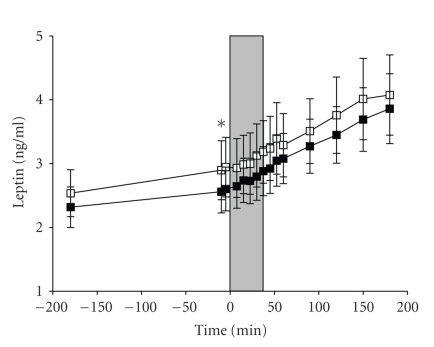
Mean (±SEM) plasma leptin concentrations in the hypoxic (white squares) and the control (black squares) condition. Gray area marks the time of hypoxic/normoxic intervention. The red label marks the point of time leptin has significantly increased. The time course describes the period before and after the hypoxic intervention was initiated (Time point 0).

Figure 2 shows the course of leptin concentrations during normoxic and hypoxic conditions under euglycemia. Overall, leptin levels increased significantly during hypoxia (*P* < .001) and normoxia (*P* < .001) in the overall time course of 6 hours of measurements. There were no statistical differences in leptin levels between hypoxic and normoxic conditions as revealed by ANOVA for repeated measurements for the “session” effect (*P* = .269) and the interaction effect (*P* = .638). Analyses of the AUCs of leptin concentrations confirmed that there was no difference between the two conditions (*P* = .180). Paired *t*-test comparison revealed that there were no differences between the groups at baseline (*P* = .446). Similarly, after having reached the euglycemic equilibrium, plasma leptin concentrations showed no significant differences between conditions directly before the induction of hypoxia, during the hypoxic period, and during the 2.5 hours period after hypoxia in *t*-test comparison of single time points (*P* = .137 for all).

## 4. Discussion

Our results show that plasma leptin concentrations are not affected by acute hypoxia as compared to normoxic control in healthy subjects at rest. In contrast, previous studies showed an inverse correlation of serum leptin levels to altitude [11, 19, 20], that is, when altitude increase serum leptin levels drop. However the effects of hypoxia on circulating leptin concentrations are contradicting as recently discussed in detail by Sierra-Johnson et al. [21–23].

The underlying cause why our results do not confirm previous data indicating an increasing effect of hypoxia on leptin levels deserves to be explained. One reasoning may be based on hormonal influences. Norepinephrine and epinephrine have been shown to inhibit leptin secretion [24–26], whereas glucocorticoids, for example, dexamethasone, stimulate leptin secretion in humans [27, 28]. So it may be speculated that in our study a potential increase in leptin concentrations upon hypoxia may be masked by suppressing effects of the risen epinephrine levels under this condition. This mechanism, however, can only remain speculation within the scope of an in vivo approach as applied here and requires some confirmation in further studies.

Our second finding was a significant increase in leptin concentrations in the course of the six hours of the experimental procedure. This is in line with the finding that circulating leptin concentrations display ultradian and circadian rhythmicity with a nadir about noon and the early afternoon, and peak levels during the night [29, 30]. The increase in leptin concentrations starting prenoon and the following 6 hours as reported here may reflect this diurnal variation. Moreover, the increased leptin concentrations may also be due to the standardized insulin infusion during the clamp [16, 17, 31]. Since insulin has been shown to elevate leptin concentrations [17, 32], we intended to exclude this biasing factor by applying the hyperinsulinemic-euglycemic clamp technique which adjusts constant insulin concentrations during the experiments. Notwithstanding, this method may have contributed to the overall rise in leptin levels even though in all conditions equally. 

One central question we intended to focus by our study was if alterations in leptin concentrations in OSAS patients could be assigned either to recurrent hypoxia or to the known high BMI which is a frequently found feature in this disease. Generally, leptin is produced by white adipose tissue and downregulates food intake by inhibiting neuropeptide Y synthesis and elevating alpha-melanocyte stimulating hormone synthesis in the hypothalamus, a feedback mechanism signalling increased peripheral fat reserves to cerebral appetite centers [33]. Indeed, it has been shown that serum leptin levels are positively correlated with the BMI [33, 34]. Remarkably, some studies could demonstrate that OSAS patients, however, display higher leptin levels than BMI-matched obese and normal weight subjects [34, 35]. Since Leptin is a physiological feedback signal to reduce appetite and thereby should prevent the development of obesity, the occurrence of overweight and elevated circulating leptin concentrations, as seen in OSAS patients, deserves an explanation. There are two possible mechanisms conceivable. One possible explanation is a congenital leptin deficiency, although this is a rarely seen defect [36]. The second avowal could be a relative leptin resistance, due to the fact that leptin seems to downregulate its own receptors, so high leptin levels could possibly reduce the number of leptin receptors and generate a relative leptin resistance which in turn causes obesity [37]. On the other hand, it was demonstrated that the apnea-hypopnea index (AHI) correlates positively with serum leptin concentrations in patients with OSAS but not in the BMI-matched control group and there was a significant decrease of serum leptin levels in OSAS patients upon treatment with CPAP therapy over six months despite of stable BMI values [34, 38]. Therefore, we can speculate that recurrent oxygen desaturations in OSAS patients may play a major role in the regulation of leptin concentrations disregarding the influence of obesity. Notwithstanding, our data do not confirm the hypothesis that elevated leptin concentrations in OSAS patients are caused by hypoxia either. This negative result, however, may be based on our study protocol which fundamentally differs from the approach of other investigations. Our subjects underwent only a single moderate hypoxic episode of 30 minutes in length. This hypoxic pattern differs from previous studies according to length, frequency, and degree of oxygen desaturation [8, 9, 11, 12]. Zaccaria et al., for example, studied male subjects at high altitude with an observation period of 20 days [11]. Therefore, it remains to be carefully considered to what extent our findings can be assigned to the hypoxic conditions as present in OSAS where severe hypoxic periods, displaying distinctly lower oxygen saturation values than 75%, can be observed [8]. So eventually our negative findings may simply reflect an insufficient duration and the extent of hypoxia to affect leptin secretion. Notwithstanding, our study design has been established to induce clinically relevant changes in other features of the metabolic syndrome [2, 3] and is therefore an adequate approach to gain first insight into the effects of acute hypoxia in this context in humans. In addition, this is the first study investigating the effects of acute hypoxia on leptin concentrations controlling for fundamental influencing factors in healthy men. According to our data, plasma leptin levels seem to be unaffected by short hypoxic episodes of moderate degree. Future studies are desirable to further clarify the relationship between hypoxia and leptin regulation in physiological and pathophysiological states. 

## Figures and Tables

**Figure 3:**
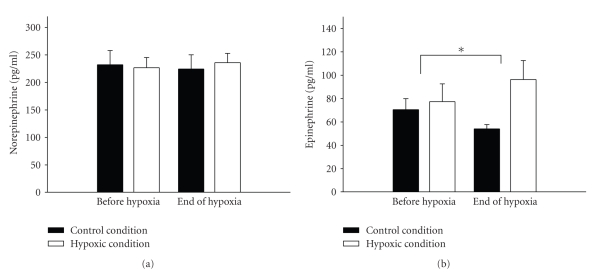
Mean (±SEM) norepinephrine concentrations (a) in the hypoxic (white bars) and the control (black bars) condition. Mean (±SEM) epinephrine concentrations (b) in the hypoxic (white bars) and the control (black bars) condition. Epinephrine concentrations have significantly increased in the hypoxic condition.

## References

[1] Hosogai N, Fukuhara A, Oshima K (2007). Adipose tissue hypoxia in obesity and its impact on adipocytokine dysregulation. *Diabetes*.

[2] Oltmanns KM, Gehring H, Rudolf S (2004). Hypoxia causes glucose intolerance in humans. *American Journal of Respiratory and Critical Care Medicine*.

[3] Oltmanns KM, Gehring H, Rudolf S (2006). Persistent suppression of resting energy expenditure after acute hypoxia. *Metabolism*.

[4] Oltmanns KM, Gehring H, Rudolf S (2006). Acute hypoxia decreases plasma VEGF concentration in healthy humans. *American Journal of Physiology*.

[5] Hubold C, Oltmanns KM, Schultes B (2006). High plasma VEGF relates to low carbohydrate intake in patients with type 2 diabetes. *International Journal of Obesity*.

[6] Auwerx J, Staels B (1998). Leptin. *The Lancet*.

[7] Chin K, Shimizu K, Nakamura T (1999). Changes in intra-abdominal visceral fat and serum leptin levels in patients with obstructive sleep apnea syndrome following nasal continuous positive airway pressure therapy. *Circulation*.

[8] Harsch IA, Konturek PC, Koebnick C (2003). Leptin and ghrelin levels in patients with obstructive sleep apnoea: effect of CPAP treatment. *European Respiratory Journal*.

[9] Tschöp M, Strasburger CJ, Hartmann G, Biollaz J, Bärtsch P (1998). Raised leptin concentrations at high altitude associated with loss of appetite. *The Lancet*.

[10] Snyder EM, Carr RD, Deacon CF, Johnson BD (2008). Overnight hypoxic exposure and glucagon-like peptide-1 and leptin levels in humans. *Applied Physiology, Nutrition and Metabolism*.

[11] Zaccaria M, Ermolao A, Bonvicini P, Travain G, Varnier M (2004). Decreased serum leptin levels during prolonged high altitude exposure. *European Journal of Applied Physiology*.

[12] Meißner U, Hänisch C, Östreicher I (2005). Differential regulation of leptin synthesis in rats during short-term hypoxia and short-term carbon monoxide inhalation. *Endocrinology*.

[13] Grosfeld A, Zilberfarb V, Turban S, André J, Guerre-Millo M, Issad T (2002). Hypoxia increases leptin expression in human PAZ6 adipose cells. *Diabetologia*.

[14] Yasumasu T, Takahara K, Nakashima Y (2002). Hypoxia inhibits leptin production by cultured rat adipocytes. *Obesity Research*.

[15] Raff H, Bruder ED, Jankowski BM, Colman RJ (2001). Effect of neonatal hypoxia on leptin, insulin, growth hormone and body composition in the rat. *Hormone and Metabolic Research*.

[16] Fruehwald-Schultes B, Oltmanns KM, Kern W, Born J, Fehm HL, Peters A (2002). The effect of experimentally induced insulin resistance on the leptin response to hyperinsulinaemia. *International Journal of Obesity*.

[17] Wellhoener P, Fruehwald-Schultes B, Kern W (2000). Glucose metabolism rather than insulin is a main determinant of leptin secretion in humans. *The Journal of Clinical Endocrinology & Metabolism*.

[18] Chan JL, Wong SL, Orlova C, Raciti P, Mantzoros CS (2007). Pharmacokinetics of recombinant methionyl human leptin after subcutaneous administration: variation of concentration-dependent parameters according to assay. *The Journal of Clinical Endocrinology & Metabolism*.

[19] de León AC, González DA, Pérez Méndez LI (2004). Leptin and altitude in the cardiovascular diseases. *Obesity Research*.

[20] Vats P, Singh VK, Singh SN, Singh SB (2007). High altitude induced anorexia: effect of changes in leptin and oxidative stress levels. *Nutritional Neuroscience*.

[21] Raff H (2008). Control of leptin with altitude exposure. *Journal of Applied Physiology*.

[22] Sierra-Johnson J, Romero-Corral A, Somers VK, Johnson BD (2008). Last word on viewpoint: effect of altitude on leptin levels, does it go up or down?. *Journal of Applied Physiology*.

[23] Sierra-Johnson J, Romero-Corral A, Somers VK, Johnson BD (2008). Effect of altitude on leptin levels, does it go up or down?. *Journal of Applied Physiology*.

[24] Gettys TW, Harkness PJ, Watson PM (1996). The *β*
_3_-adrenergic receptor inhibits insulin-stimulated leptin secretion from isolated rat adipocytes. *Endocrinology*.

[25] Li H, Matheny M, Scarpace PJ (1997). *β*
_3_-adrenergic-mediated suppression of leptin gene expression in rats. *American Journal of Physiology*.

[26] Fritsche A, Wahl H-G, Metzinger E (1998). Evidence for inhibition of leptin secretion by catecholamines in man. *Experimental and Clinical Endocrinology and Diabetes*.

[27] Papaspyrou-Rao S, Schneider SH, Petersen RN, Fried SK (1997). Dexamethasone increases leptin expression in humans in vivo. *Journal of Clinical Endocrinology and Metabolism*.

[28] Dagogo-Jack S, Selke G, Melson AK, Newcomer JW (1997). Robust leptin secretory responses to dexamethasone in obese subjects. *The Journal of Clinical Endocrinology & Metabolism*.

[29] Sinha MK, Ohannesian JP, Heiman ML (1996). Nocturnal rise of leptin in lean, obese, and non-insulin-dependent diabetes mellitus subjects. *The Journal of Clinical Investigation*.

[30] Randeva HS, Karteris E, Lewandowski KC, Sailesh S, O'Hare P, Hillhouse EW (2003). Circadian rhythmicity of salivary leptin in healthy subjects. *Molecular Genetics and Metabolism*.

[31] Saad MF, Khan A, Sharma A (1998). Physiological insulinemia acutely modulates plasma leptin. *Diabetes*.

[32] Stefan N, Fritsche A, Häring H, Stumvoll M (2001). Acute stimulation of leptin concentrations in humans during hyperglycemic hyperinsulinemia. Influence of free fatty acids and fasting. *International Journal of Obesity*.

[33] Ciftci TU, Kokturk O, Bukan N, Bilgihan A (2005). Leptin and ghrelin levels in patients with obstructive sleep apnea syndrome. *Respiration*.

[34] Vgontzas AN, Papanicolaou DA, Bixler EO (2000). Sleep apnea and daytime sleepiness and fatigue: relation to visceral obesity, insulin resistance, and hypercytokinemia. *The Journal of Clinical Endocrinology & Metabolism*.

[35] Drummond M, Winck JC, Guimarães JT, Santos AC, Almeida J, Marques JA (2008). Autoadjusting-CPAP effect on serum leptin concentrations in obstructive sleep apnoea patients. *BMC Pulmonary Medicine*.

[36] Gibson WT, Farooqi IS, Moreau M (2004). Congenital leptin deficiency due to homozygosity for the Δ133G mutation: report of another case and evaluation of response to four years of leptin therapy. *The Journal of Clinical Endocrinology & Metabolism*.

[37] Martin SS, Qasim A, Reilly MP (2008). Leptin resistance: a possible interface of inflammation and metabolism in obesity-related cardiovascular disease. *Journal of the American College of Cardiology*.

[38] Ip MSM, Lam KSL, Ho C-M, Tsang KWT, Lam W (2000). Serum leptin and vascular risk factors in obstructive sleep apnea. *Chest*.

